# Supplementary material for: Structure of Mycobacterium tuberculosis phosphatidylinositol phosphate synthase reveals mechanism of substrate binding and metal catalysis
Source: Commun Biol. 2019 May 8;2:175. doi: 10.1038/s42003-019-0427-1 (PMC6506517; doi:10.1038/s42003-019-0427-1)
Supplement: Supplementary file 3 — Supplementary Information [file 42003_2019_427_MOESM3_ESM.pdf]

Supplementary Figures

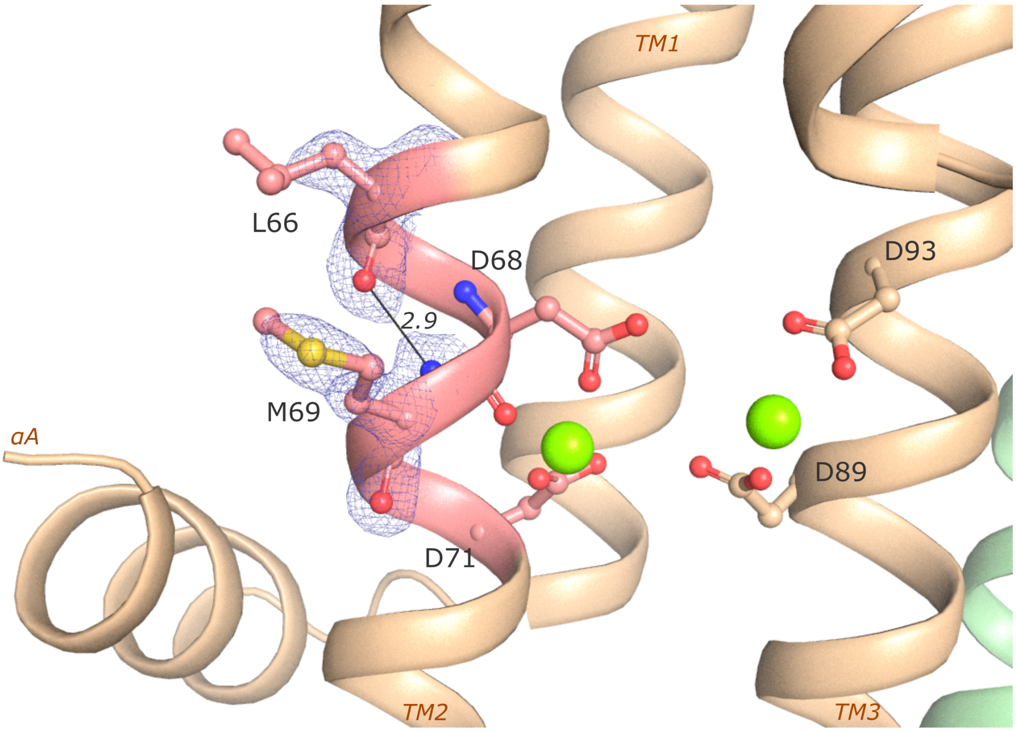

Supplementary Figure 1.
3_{10} helix motif in the *M. tuberculosis* PgsA1. $2F_o$-F_c electron density map around L66 and M69 is contoured at 1.5 σ. The black line indicates the $i + 3 \rightarrow i$ hydrogen bond defining the 3_{10} helix motif (pink). The length of the hydrogen bond is indicated in Å.

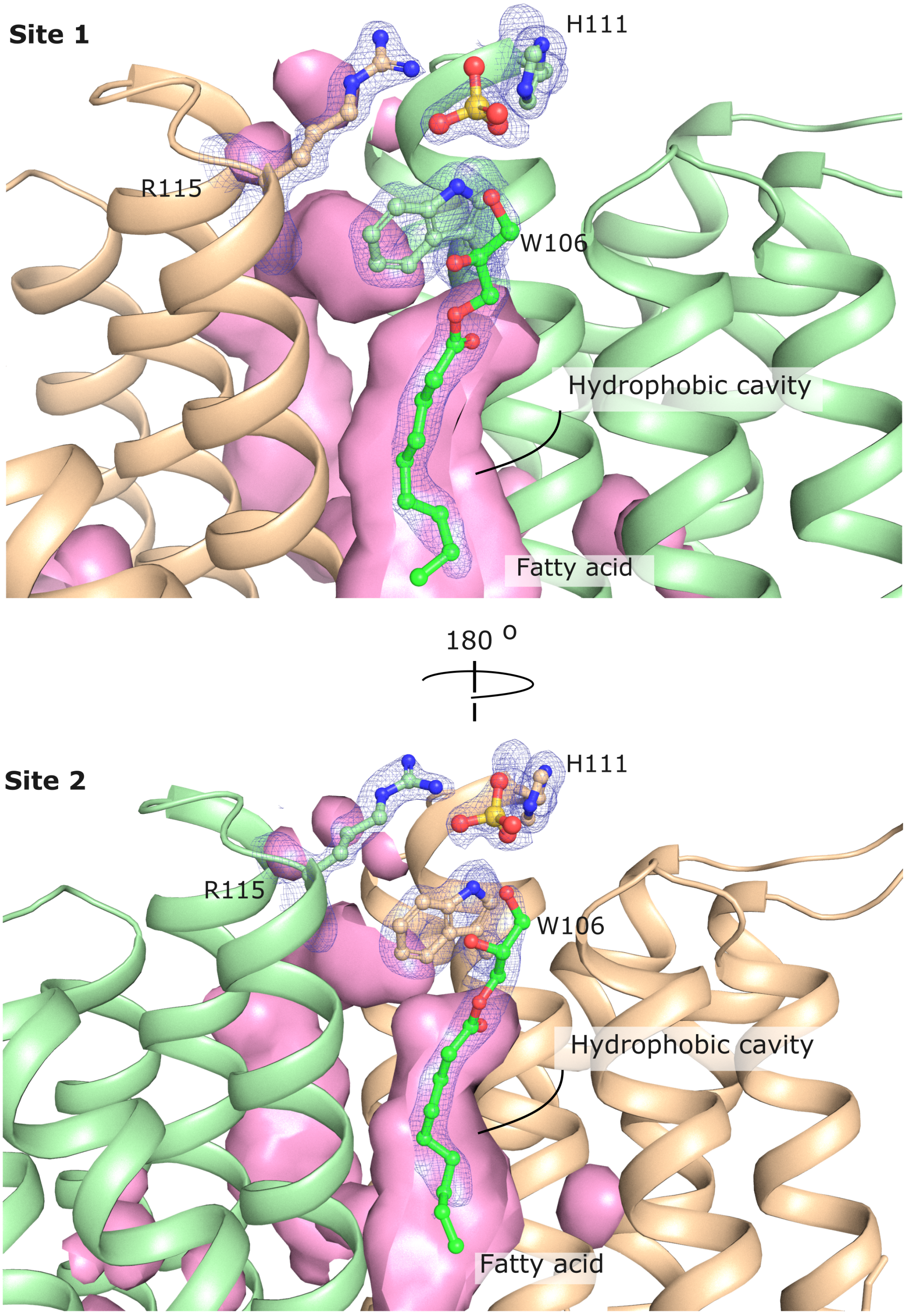

Supplementary Figure 2. Potential membrane phospholipid binding sites.
Fragment of an unidentified lipid molecule, bound to the large hydrophobic cavity on a dimer interface, was modeled as a 2,3-Dihydroxypropyl fatty acid (monoolein fragment). Cavities (pink) were identified and visualized using PyMOL software (version 2.0.0).
$2F_o$-F_c electron density map (blue mesh) is contoured at 1.5 σ.

```
O29976 DIPPS_ARCFU     301   RKINRKISTRISAAIVNKVNPNQMTLISFLVG------------AFSALASFFSIP----   344
P0ABF8 PGSA_ECOLI        1   ---------------MQFNIPTLLTLFRVILIPFF------------VLVFYLPVTWSPF    33
Q8VDP6 CDIPT_MOUSE       1   ----------MPEENIFLFVPNLIGYARIVFA---------------IIS-FYFMPCCPF    34
P9WPG7 PISA_MYCTU        3   -----KLPFLSRAAFARITTPIARGLLRVGLTPDVVTILGTTASVAGALT-LFPMG-KLF    55
P06197 PIS_YEAST         3   ---SNSTPEKVTAEHVLWYIPNKIGYVRVITA---------------ALS-FFVMKNHPT    43
                                                 *       .                   :     :

O29976 DIPPS_ARCFU     345   LAGLLYQFSSILDGCDGEIARASLKMSKKGGYVDSILDRFVDFLFLAIIALLYPKTATVA   404
P0ABF8 PGSA_ECOLI       34   AAALIFCVAAVTDWFDGFLARRWNQSTRFGAFLDPVADKVLVAIAMVLVTEHYHSWWVTL    93
Q8VDP6 CDIPT_MOUSE      35   TASSFYLLSGLLDAFDGHAARALNQGTRFGAMLDMLTDRCATMCLLVNLALLYPRATLLF    94
P9WPG7 PISA_MYCTU       56   AGACVVWFFVLFDMLDGAMARERGGGTRFGAVLDATCDRISDGAVFCGL-----LWWIAF   110
P06197 PIS_YEAST        44   AFTWLYSTSCLLDALDGTMARKYNQVSSLGAVLDMVTDRSSTAGLMCFLCVQYPQWCVFF   103
                                  .     : *  **  **     :  *. :*   *:      :  :

O29976 DIPPS_ARCFU     405   MFAIF--GSVMVSYTSEKYKAEFGESIFGK--FRVLNYIP--------------GKRDER   446
P0ABF8 PGSA_ECOLI       94   PAATMIAREIIIS-ALREWMAEL-----GKRSSVAVSWIGKVKTTAQMVALAWLLWRPNI   147
Q8VDP6 CDIPT_MOUSE      95   QLSMS------LD-VASHWLHLHSSVVRGSESHKMI---D---LSGNPVLRIYYTSRPAL   141
P9WPG7 PISA_MYCTU      111   HMRDR---PLVIA-TL---ICLVT--------SQVISYIKARAEASGLRGDGGFIERPER   155
P06197 PIS_YEAST       104   QLMLG------LD-ITSHYMHMYASLSAGKTSHKSV---G---EGESRLLHLYYTRRDVL   150
                                         :                       :                    *

O29976 DIPPS_ARCFU     447   IFLI-----MIFCLLSA---ISLQWIFWMF-----LFVAAISLTRVVVT-LLAVLVSK--   490
P0ABF8 PGSA_ECOLI      148   W-VEYAGIALFFVAAVLTLWSMLQYLS--------------------------------A   174
Q8VDP6 CDIPT_MOUSE     142   F-TLCAGNELFYCLLYLFNFSEGPLVGSVGLFRMGLWVTA---PIALLKSVISVIHLITA   197
P9WPG7 PISA_MYCTU      156   LIIVLTGA----------GVSDFPFVPWPPALSVGMWLLAVASVITCVQRLHTVWTSPGA   205
P06197 PIS_YEAST       151   F-TICAFNELFYAGLYLQLFSNSATFGK--------WTTIISFPGYVFKQTANVVQLKRA   201
                                                      .

O29976 DIPPS_ARCFU     491   -------------------                                           490
P0ABF8 PGSA_ECOLI      175   ARA----DLLDQ-------                                           182
Q8VDP6 CDIPT_MOUSE     198   ARNMAALDAADRAKKK---                                           213
P9WPG7 PISA_MYCTU      206   IDRMAIPGKGDR-------                                           217
P06197 PIS_YEAST       202   ALILADNDAKNANEKNKTY                                           220
```

Supplementary Figure 3. Alignment of representative sequences from CDP-alcohol phosphotransferase family (PF01066). Amino acids are coloured by similarity.

O29976: Bifunctional IPC transferase and DIPP synthase (gene *AF_0263*) from *Archaeoglobus fulgidus.* Sequence corresponds to DIPPS domain; the rest of the sequence was truncated.

P0ABF8: CDP-diacylglycerol-glycerol-3-phosphate 3-phosphatidyltransferase (gene *pgsA*) from *Escherichia coli.*

Q8VDP6: CDP-diacylglycerol-inositol 3-phosphatidyltransferase (gene *Cdipt*) from *Mus musculus* (mouse).

P9WPG7: CDP-diacylglycerol-inositol 3-phosphatidyltransferase (gene *pgsA1*) from *Mycobacterium tuberculosis*.

P06197: CDP-diacylglycerol-inositol 3-phosphatidyltransferase (gene *PIS1*) from *Saccharomyces cerevisiae* (Baker's yeast)

Sequence alignment generated by Clustal Omega sequence alignment tool[1].

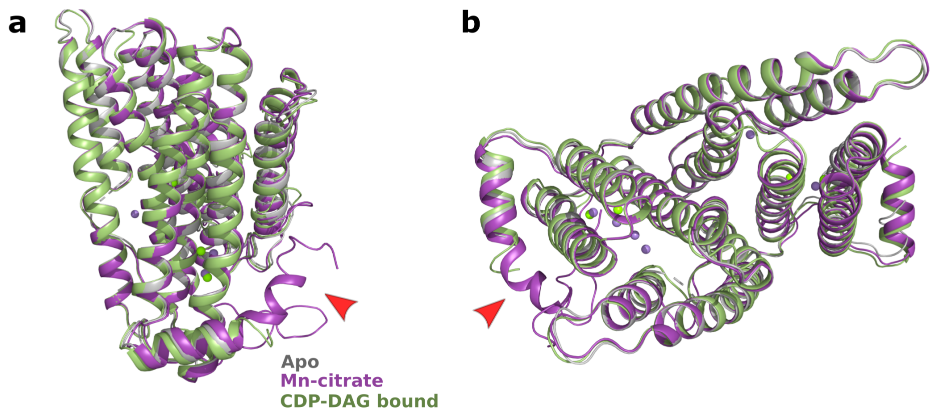

Supplementary Figure 4. Structural alignment of apo, Mn-citrate bound and CDP-DAG bound *M. tuberculosis* PgsA1 crystal structures.
(a) Side view. **(b)** Top view. Red arrows indicate differences in structure of N-terminal amphipathic helix.

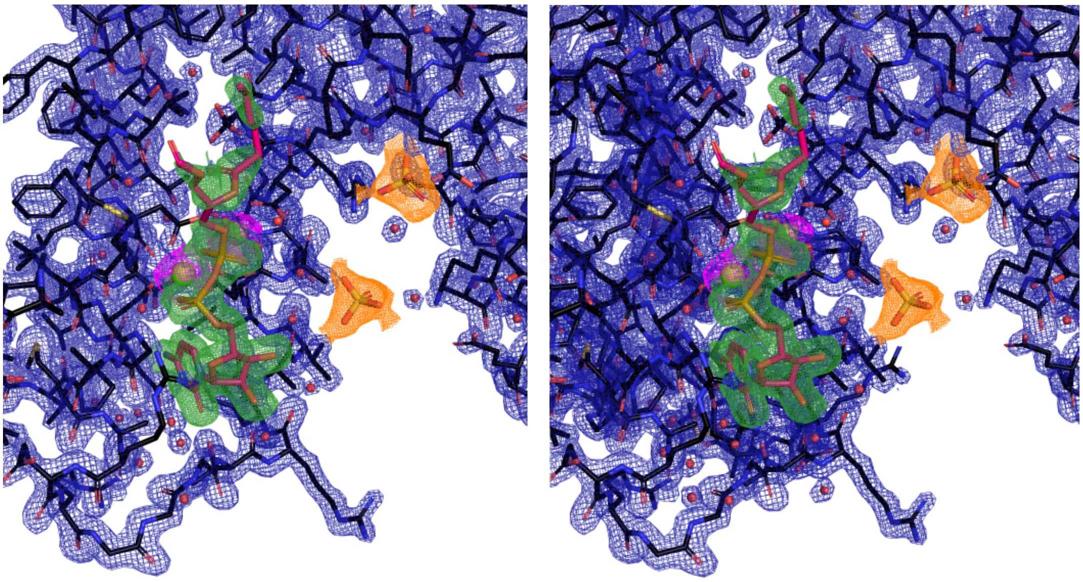

Supplementary Figure 5. Wall-eye stereo view of electron density around the CDP-DAG binding site in the *M. tuberculosis* PgsA1 chain A.
The electron density maps are generated using final refined model of the CDP-DAG bound *M. tuberculosis* PgsA1. The final $2F_o\text{-}F_c$ map (blue mesh) around the polypeptide chain is contoured at 1.5σ; An omit map for the CDP-DAG is contoured at 2.5σ and shown as a green mesh, an omit map for the Mg ions is contoured at 1.5σ and shown as a magenta mesh, an omit map for the SO_4 ions is contoured at 1.5σ and shown as an orange mesh. Protein chain B, as well as the other bound ligands were hidden for clarity.

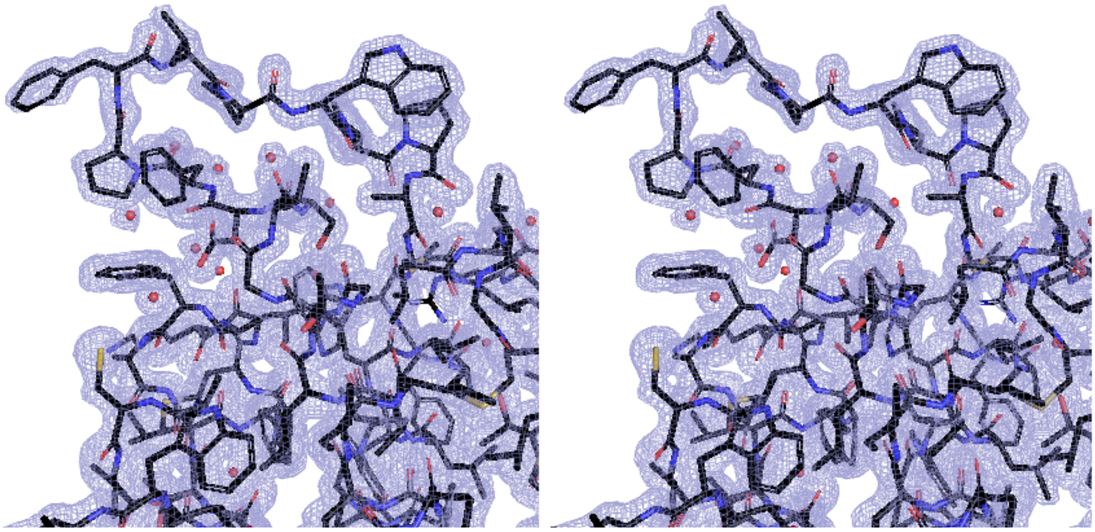

Supplementary Figure 6. Wall-eye stereo view of electron density around a selected amino acid stretch in the *Mt* PgsA1 chain A.
The electron density map is generated using final refined model of the CDP-DAG bound *M. tuberculosis* PgsA1. The final $2F_o$-F_c map (blue mesh) around the polypeptide chain is contoured at 1.5σ. Water molecules are shown as spheres. Protein chain B, as well as the other bound ligands were hidden for clarity.

Supplementary Tables

Supplementary Table 1. Sequences of the oligonucleotides used for cloning of *M. tuberculosis pgsA1* and site-directed mutagenesis

Oligo Name	*Sequence, 5' - 3'*
Rv2612c_cLIC_Forward	ATGGGTGGTGGATTTGCTagcaagctgcccttcctgtccc
Rv2612c_cLIC_Reverse	TTGGAAGTATAAATTTTCccggtcgccctttccaggaatc
A90Y_Forward	gcgcggtgctggacTATacctgtgaccgcatcagtgacg
A90Y_Reverse	cgtcactgatgcggtcacaggtATAgtccagcaccgcgc
R94K_Forward	ctggacgccacctgtgacAAAatcagtgacggcgcg
R94K_Reverse	cgcgccgtcactgatTTTgtcacaggtggcgtccag
R94Q_Forward	gacgccacctgtgacCAGatcagtgacggcg
R94Q_Reverse	cgccgtcactgatCTGgtcacaggtggcgtc
Y133F_Forward	tcgcaggtgatctctTTTatcaaggcccgggcg
Y133F_Reverse	cgcccgggccttgatAAAagagatcacctgcga
Y133E_Forward	ctcgcaggtgatctctGAAatcaaggcccgggcgg
Y133E_Reverse	ccgcccgggccttgatTTCagagatcacctgcgag
R137K_Forward	ctcttacatcaaggccAAAgcggaggccagcgggc
R137K_Reverse	gcccgctggcctccgcTTTggccttgatgtaagag
R137Q_Forward	ttacatcaaggccCAGgcggaggccagc
R137Q_Reverse	gctggcctccgcCTGggccttgatgtaa

Cloning primers include 5' extensions indicated in capital letters necessary for ligation independent cloning (LIC). Three capital letters in primers used for site-directed mutagenesis indicate introduced point mutations.

Supplementary references

1. Sievers F, Wilm A, Dineen DG, Gibson TJ, Karplus K, Li W, Lopez R, McWilliam H, Remmert M, Söding J, Thompson JD, Higgins DG. Fast, scalable generation of high-quality protein multiple sequence alignments using Clustal Omega. *Molecular Systems Biology* 7:539 (2011). doi:10.1038/msb.2011.75
