# Supplementary material for: Structure of Mycobacterium tuberculosis phosphatidylinositol phosphate synthase reveals mechanism of substrate binding and metal catalysis
Source: Commun Biol. 2019 May 8;2:175. doi: 10.1038/s42003-019-0427-1 (PMC6506517; doi:10.1038/s42003-019-0427-1)
Supplement: Supplementary file 4 — Description of Additional Supplementary Files [file 42003_2019_427_MOESM4_ESM.pdf]

Description of additional supplementary files

Supplementary Movies 1and 2. The CDP-DAG bound *M. tuberculosis* PgsA1 structure reveal an outward movement and rotation of TM2 along its axis and subsequent slide of αA along the TM2, as compared to the apo structure.

Supplementary Movie 1: Zoomed in view on 3_{10} helix motif of the *M. tuberculosis* PgsA1, chain B. The CDP-DAG bound *M. tuberculosis* PgsA1 structure is shown in cyan and the apo structure – in silver.

Supplementary Movie 2: A side view of the αA in the *M. tuberculosis* PgsA1, chain B. The CDP-DAG bound *M. tuberculosis* PgsA1 structure is shown in cyan and the apo structure – in silver.

Videos are prepared in PyMOL software (version 2.0.0).

Supplementary Data 1. Source data used for generation of figure 5b.
